# FGF19 Is Coamplified With CCND1 to Promote Proliferation in Lung Squamous Cell Carcinoma and Their Combined Inhibition Shows Improved Efficacy

**DOI:** 10.3389/fonc.2022.846744

**Published:** 2022-04-07

**Authors:** Yanshuang Zhang, Tingyu Wu, Fan Li, Yirui Cheng, Qing Han, Xin Lu, Shun Lu, Weiliang Xia

**Affiliations:** ^1^State Key Laboratory of Oncogenes and Related Genes, Ren Ji Hospital, School of Medicine and School of Biomedical Engineering, Shanghai Jiao Tong University, Shanghai, China; ^2^Shanghai Lung Cancer Center, Shanghai Chest Hospital, Shanghai Jiao Tong University, Shanghai, China

**Keywords:** LUSC, FGF19, FGFR4, CCND1, CDK4/6, amplification, combined inhibition

## Abstract

Lung squamous cell carcinoma (LUSC) remains as a major cause of cancer-associated mortality with few therapeutic options. Continued research on new driver genes is particularly important. FGF19, a fibroblast growth factor, is frequently observed as amplified in human LUSC, which is also associated with multiple genomic gains and losses. However, the importance of these associated changes is largely unknown. In this study, we aimed to clarify a novel mechanism that link neighboring oncogene co-amplification in the development of LUSC. We found that FGF19 was co-amplified and co-expressed with its neighboring gene CCND1 in a subset of LUSC patients and associated with poor prognosis. Moreover, FGF19 combined with CCND1 promoted the cell cycle progression of LUSC cells. Mechanistically, FGF19 also enhanced CCND1 expression by activating FGFR4-ERK1/2 signaling and strengthening CCND1-induced phosphorylation and inactivation of retinoblastoma (RB). In a murine model of lung orthotopic cancer, knockdown of CCND1 was found to prolong survival by attenuating FGF19-induced cell proliferation. Furthermore, the combination treatment of the FGFR4 inhibitor BLU9931 and the CDK4/6 inhibitor palbociclib potentiated the growth inhibition and arrested cells in G1 phase. *In vivo*, co-targeting FGFR4 and CDK4/6 also showed marked inhibition of tumor growth than single agent treatment. These findings further elucidate the oncogenic role of FGF19 in LUSC and provide insights into how the co-amplification of neighboring genes synergistically function to promote cancer growth, and combined inhibition against both FGF19 and CCND1 is more effective.

## Introduction

Lung cancer is the leading cause of cancer-related death around the world (1, 2). The therapy of lung adenocarcinoma (LUAD), especially the targeted therapy against driver genes, has made significant progress, but patients with lung squamous cell carcinoma (LUSC) have benefited little from targeted therapy (3, 4). Thus, it is essential to search for potential driving factors/genes in LUSC and identify the effectiveness of targeted therapy and propose more effective treatment schemes.

Amplification of chromosomal regions can play important roles in carcinogenesis. Amplification of specific chromosomal sites not only affect individual genes, but also cause the overexpression of neighboring genes (5). The amplified neighboring genes may cooperate to promote tumor initiation and progression, and the relevant mechanism depends on the molecular relationship between the neighboring genes themselves (6, 7). Understanding the cooperative mechanism of co-amplified genes may help formulate new therapeutic strategies for lung cancer. Our previous studies have found that the amplified region 11q13.3 containing fibroblast growth factor 19 (FGF19) and cyclin D1 (CCND1) in smoking-LUSC patients appear frequently. However, whether they have a synergistic interaction has not been further explored (8).

FGF signals regulate various biological processes during development and adulthood through FGF receptor (FGFR) tyrosine kinases (9). The FGF19 subfamily, particularly, FGF19, FGF21, and FGF23, acts as hormones or endocrine factors that bind to specific receptors (10). Under normal circumstances, FGF19 is secreted from the intestinal tract, and binds to FGFR4 on liver cells through the hepatoenteral circulation to regulate a variety of metabolic processes, namely, the metabolism of glucose, lipid, and bile acid, and gallbladder filling (11–13). In disease states, FGF19 is crucial for the development and progression of several cancers such as head and neck squamous cell carcinoma (14), hepatocellular carcinoma (15), and lung cancer (16). The beginning of cell division integrates a large amount of intracellular and extracellular inputs. Cyclin D connects these inputs to the start of DNA replication (17). Under the stimulation of extracellular signals like mitogens, cytokines, cell–cell contacts and differentiation inducers, cyclin D promotes cell division by activating CDK4/6, which in turn phosphorylates the retinoblastoma (RB) tumor suppressor leading to unrestrained E2F transcription factors, E2F-dependent transcription activation and progression through G1 to S phase (18, 19). Therefore, the increase in the level and activity of the cyclin D-CDK4/6 complex is closely related to uninhibited cell proliferation and cancer (20, 21). CCND1, a crucial member of the CCND family, is an established human oncogene. There has been a substantial evidence that CCND1 involves in a variety of cancers, namely, breast cancer, lung cancer, melanoma, and oral squamous cell carcinoma (22).

Here, we demonstrated with more extensive and sufficient data that FGF19 frequently co-amplified with its neighboring gene CCND1 in LUSC. CCND1 expression was also upregulated by FGF19 through the FGF19–FGFR4–ERK1/2 signaling pathway. FGF19 enhanced CCND1-induced RB phosphorylation and promoted cell cycle progression. Knockdown of CCND1 was found to prolong survival by attenuating FGF19-induced cell proliferation in a murine lung cancer model. In addition, co-targeting FGFR4 and CDK4/6 could significantly inhibit FGF19-driven LUSC proliferation and tumor growth *in vitro* and in mouse models. Thus, these findings further elucidate the oncogenic role of FGF19 in LUSC and provide insights into how the co-amplification of neighboring genes synergistically function to promote cancer growth, and combined inhibition against both genes is more effective.

## Materials and Methods

### Clinical Tissue Samples

Sixteen human LUSC tissue samples were obtained from the Shanghai Chest Hospital from January 2017 to November 2017. Our previous work had summarized all the clinical pathological characteristics of the samples (23). The study is approved by the Research Ethics Committee of the School of Biomedical Engineering, Shanghai Jiao Tong University.

### Cell Culture and Reagents

H520, SK-MES-1, HCC95, and H1703 were purchased from the American Type Culture Collection (ATCC). H520, HCC95, and H1703 cultured using 1640 (HyClone) and SK-MES-1 cultured using MEM (HyClone) with non-essential amino acids solution (Gibco, 11140050) and Sodium Pyruvate (Gibco, 11360070). All medium supplemented with 10% fetal bovine serum (Lonsera, S711-001S), 100 U/ml penicillin, and 100 μg/ml streptomycin (Hyclone, SV30010). All cell lines were grown at 37°C with a humidified 5% CO_2_ atmosphere. Recombinant human FGF19 protein (R&D SYSTEMS, 969-FG) was used to activate FGFR4. All inhibitor were purchased from the MedChemExpress and dissolved in DMSO, and then, were aliquoted and stored as 10 mM stocks at −80°C for *in vitro* studies.

### Lentivirus Transduction and Generation of Stable Cell Lines

Human full-length FGF19 gene was amplified from ORF plasmids (OriGene, #RC203750) and cloned into pCDH-CMV-MCS-EF1-copGFP backbone (Addgene plasmid, #72265) to construct plasmid for lentivirus production. HEK293T cells were transfected with FGF19-overexpression plasmid or empty vector, accompanied by pCMV-VSV-G/pCMV-dR8.2 plasmids. Mature FGF19 overexpression lentivirus and control lentivirus were obtained by ultracentrifugation. FGF19 shRNA lentiviruses and CCND1 shRNA were constructed by the GENECHEM Biotechnology Co. Ltd. (Shanghai, China). Cells were transfected with lentivirus in 24-well plate at 50% confluency, and then stably transfected cells were sorted by flow cytometry.

### Western Blot, RNA Extraction, cDNA Synthesis, and Quantitative Real-Rime PCR (qRT-PCR) Analysis

Cells were lysed for protein or RNA extraction, and subjected to western blot or cDNA synthesis and qRT-PCR was as previously described (24). Antibodies for western blot were as follows: β-Actin (Proteintech,66009-1-Ig), β-Tubulin **(**Proteintech, 66240-1-Ig**)**, FGF19 (R&D System, AF969), FGFR4 (R&D System, MAB6852), FRS2 (Abcam, ab10425), pFRS2 (R&D System, AF5126), goat anti-mouse IgG-HRP (Jackson ImmunoResearch, 115-035-003), goat anti-rabbit IgG-HRP (Jackson ImmunoResearch, 111-035-003), donkey anti-Goat IgG-HRP (Sangon Biotech, D110115) and other antibodies were obtained from the Cell Signaling Technology. Primers for qPCR were as following: CCND1, forward 5′-GTCCTACTTCAAATGTGTGCAG-3′, reverse 5′-GGGATGGTCTCCTTCATCTTAG-3′; GAPDH forward 5′-GGAGCGAGATCCCTCCAAAAT-3′, reverse 5′-GGCTGTTGTCATACTTCTCATGG-3′.

### Cell Cycle Analysis

Cells were grown to a density of 90% in a 6-well plate and collected by trypsinization. Then, the cells were fixed with 70% cold ethanol at −20°C overnight. The fixed cells were washed with PBS and treated with 100 μg/mL RNase A for 30 min at 37°C, and stained with propidium iodide (PI) at 50 μg/mL in the dark for 10 min at room temperature. Subsequently, at least 10,000 cells in each sample were analyzed by flow cytometry (BD FACS Calibur). Finally, the Modfit LT 4.0 software (BD Biosciences) was used to quantify cell populations in G0/G1, S and G2/M phases.

### mRNA-seq Analysis

LUSC cell line H520 was treated with BLU9931 (1 μM), palbociclib (1 μM) or their combination for 72 h and total RNA was extracted using an RNAiso reagent (Takara, 9109) according to the onstructions of the manufacturer, followed by treatment with RNase-free DNase I to remove genomic DNA contamination. A Qubit^®^ RNA Assay Kit in Qubit^®^2.0 Flurometer (Life Technologies, CA, USA) was used to assess the quality and quantity of RNA. RNA-seq libraries were prepared using the Hieff NGS™ MaxUp Dual-mode mRNA Library Prep Kit for Illumina^®^ (YEASEN, 12301ES96) and sequenced on the HiSeq XTen sequencers (Illumina, San Diego, CA). FastQC (version 0.11.2) was used to evaluate the quality of sequenced data. The gene expression value of the transcript was calculated by StringTie (version 1.3.3b). DESeq2 (version 1.12.4) was used to determine differential gene expression and gene was considered to be significant differentially expressed if |FoldChange| >2 and q-value <0.001.

### Immunofluorescence (IF) and Immunohistochemistry Microscopy (IHC)

For IF, cells were seeded on coverslips (WHB, WHB-24-CS) in a 24-well plate. Then, immunofluorescence experiment was conducted as previously described (24). The primary antibody is Ki-67 (Abcam, ab92742). The secondary antibody is donkey anti-rabbit IgG (H + L) highly cross-adsorbed, Alexa Fluor 594 (ThermoFisher, A-21207). Stained cells were observed and photographed with a laser scanning confocal microscopy (Leica TCS SP8). IHC experiment was performed as previously described (24). The stained sections were photographed and the software Image J was used in result analysis.

### Cell Counting Kit-8 (CCK-8) Assay

Cells (2,500 in 100 μl medium) were seeded into each well (N = 5) of a 96-well plate, and changed to medium with different concentrations of BLU9931 or palbociclib on the second day. After 72 h, 10 μl CCK8 reagent (YEASEN, #40203) was added and the optical density was measured at OD450 nm with a microplate reader (BioTek) after 1–4 h incubation.

### Colony Formation Assay

Cells transfected with lentivirus were seeded in 6-well plates about 1,000–2,000 cells per well and cultured for 1–2 weeks to measure clonogenic ability. The cells were fixed with 4% PFA for 10 min and stained with 0.1% crystal violet for 20 min, and then washed with water and followed by air drying. The colonies were photographed and then quantified based on percentage of colony area per well using ImageJ software.

### Intracellular Lactate Dehydrogenase (LDH) Assay

The intracellular LDH assay was performed to measure the levels of cell survival. LDH assay was modified from Wang′s previous studies (25). In brief, cells were lysed for 15 min in lysing buffer containing 2 mM HEPES, 0.04% Triton X-100 and 0.01% BSA (pH 7.5). Then 50 μl cell lysate was mixed with 150 μl 500 mM potassium phosphate buffer (pH 7.5) containing 2.5 mM sodium pyruvate and 0.34 mM NADH. The A_340 nm_ changes were monitored over 90 s. The cell survival rate was calculated by LDH value of the samples normalized with control culture wells.

### Flow Cytometry Analysis

Cell apoptosis was measured by flow cytometry assay. Cells were treated with media containing 10% FBS ± inhibitors for 24 h. The ApoScreen Annexin V kit (Southern Biotech, Birmingham, AL, USA) was used for flow cytometry (FACS Aria II, BD Biosciences) to detect the level of apoptosis according to the protocol of the manufacturer and the data were analyzed by FlowJo software.

### β-Galactosidase Staining

Cell senescence was quantified by measuring the β-galactosidase staining assay. Cells (3 × 10^5^/well) were plated in 6-well plates in media containing 10% FBS and then treated with DMSO or 1 µM BLU9931 or 1 µM palbociclib or 1 µM BLU9931 plus 1 µM Palbociclib; and drugs were replenished every 3 days. After 7 days, cells were stained with β-galactosidase (Beyotime, RG0039) following the protocol of the manufacturer’. The cells were photographed under a light-field microscope, and β-galactosidase-positive cells were manually counted.

### *In Vivo* Subcutaneous Lung Cancer Model

All mice were raised in the specific pathogen free (SPF) animal room of the Shanghai Jiao Tong University. Cell suspension of SK-MES-1 (2 × 10^6^ cells) or H520 (1 × 10^6^ cells) were injected subcutaneously into the right flanks of BALB/C nude mice in a volume of 50 μl. Mice body weights and tumor volumes were measured every 3 days. Tumor volumes were calculated as 0.5 × length × width^2^. After the tumor volume reached 100–200 mm^3^, the mice were randomly grouped (N = 5, per group) and enrolled into treatment groups. BLU9931 (MedChemExpress, HY-12823), diluted in a 1% (v/v) solution of Tween-80 (Sigma, P1754), was given to mice at 30 mg/kg body weight orally twice a day, and Palbociclib (MedChemExpress, HY-50767), diluted in 50 nM sodium D-lactate (TargetMol, T5220) was administered daily by oral gavage at 100 mg/kg body weight. After 3 weeks of treatment, tumors were collected and photographed. The tumor was divided into 3 parts: the first was directly fixed in 4% PFA and embedded in paraffin; the second part was dissociated into single cells and resuspended in 70% ice ethanol and placed in a storage at −20°C; and the last part was were lysed for protein and placed in a storage at −80°C. All animal experiments were performed following the regulations and internal biosafety and bioethics guidelines of the MED-X Research Institute, Shanghai Jiao Tong University (Shanghai, China).

### *In Vivo* Orthotopic Lung Cancer Model and Bioluminescence Imaging (BLI)

An orthotopic lung cancer model construction method was modified from our and Peng′s previous studies (26, 27). In brief, 5-week-old male BALB/C nude mice were anesthetized by 3% tribromoethanol in sterile PBS, 100 μl per 10 g weight, and intraperitoneal injection. A 3 mm incision was sheared on the dorsal side over left lung, 0.5 cm below the scapula on mice (N = 15 per group). After separating the subcutaneous tissues and muscles, the movement of the lungs can be observed. Cell suspension of SK-MES-1 LV-FGF19 (2 × 10^6^ cells) in a total volume of 50 μl (Matrigel: PBS = 1:4) were injected directly into the left lateral lung with insulin injection syringes (29 G ∗ 12.7 mm, BD, 328421). Mice that do not die after 3 days were considered to be successful in model construction, and then the body weight and survival period of the mice were recorded. After 25 days, 3 mice from each group were randomly selected for bioluminescent analysis. Bioluminescent signal was induced by intraperitoneal injection with 150 mg/kg D-luciferin (Meilunbio, MB1834) and imaged by the IVIS Lumina III Spectrum System (Perkin-Elmer, Waltham, MA, USA) after 10 min. Then, lungs were excised and photographed, followed by HE and PCNA staining. All the above animal experiments were performed in accordance with the protocol approved by the Institutional Ethics Committee of the Shanghai Jiao Tong University.

### Analysis of Public Data Sets From the TCGA, Oncomine and Kaplan–Meier Plotter

Relative copy number and mRNA levels of FGF19 and CCND1 of the TCGA provisional LUSC were downloaded from the Oncomine database (https://www.oncomine.org) and the cBioPortal (http://www.cbioportal.org/index.do). Linear regression and Spearman correlations between mRNA levels of FGF19 and CCND1 were conducted.

Prognostic values of FGF19 and CCND1 mRNA levels were analyzed by Kaplan–Meier survival curves of NSCLC patients, using a Kaplan–Meier Plotter (www.kmplot.com/analysis) (28).

### Gene Set Enrichment Analysis (GSEA)

Gene Set Enrichment Analysis was performed using the GSEA software and the LUSC RNA-seq datasets were downloaded from the cBioPortal database (http://www.cbioportal.org/datasets), in which LUSC dataset (TCGA, Firehose Legacy) was used. The signaling pathway of the GO and KEGG datasets were ranked by the expression of FGF19 following the official user guide of the GSEA.

### Statistical Analysis

All statistical analyses were performed using the GraphPad Prism 8 software. All data were presented as mean ± SD, and the paired or unpaired Student’s t-test or ANOVA were used to analyze the statistical significance between two groups. P-values less than 0.05 was considered statistically significant.

## Results

### FGF19 and CCND1 Are Co-Amplified and Co-Expressed in Human LUSC

**Figure 1:**
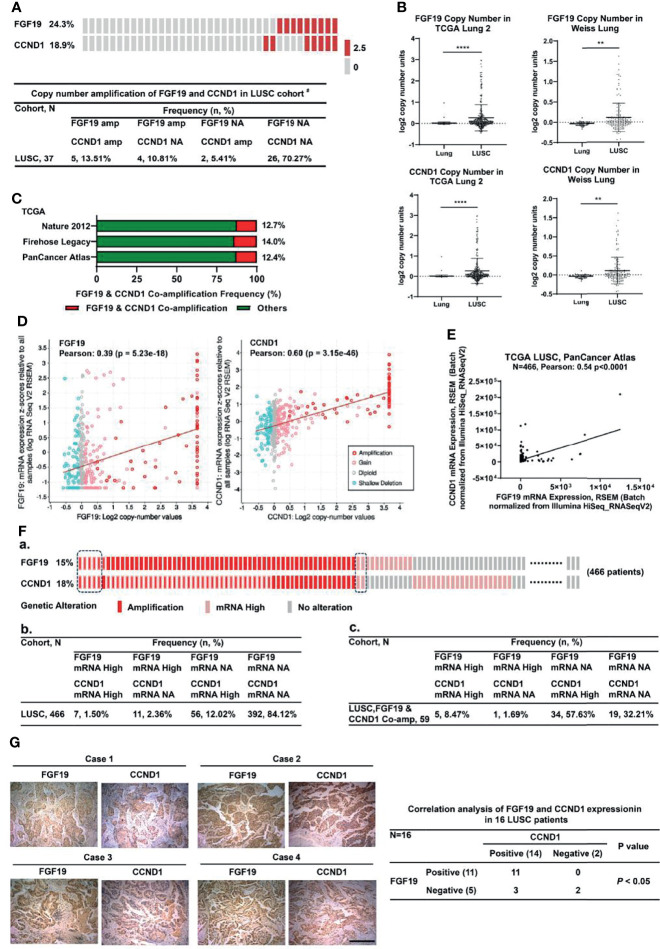
Co-amplification and co-overexpression of FGF19 and CCND1 in human LUSC. **(A)** Copy number amplification distribution of FGF19 and CCND1 in our LUSC cohort ^#^ (n = 37). Co-amplification of CCND1 is common in FGF19-amplified LUSC patient in our LUSC cohort (5/9, 55.6%). #Our previous work had summarized all the clinical pathological characteristics of the samples (23). **(B)** Amplification of FGF19 and CCND1 is common in TCGA provisional LUSC cohort. Data were shown as mean ± SD bars and compared by unpaired *t*-test. **p <0.01 and ****p <0.0001. **(C)** Co-amplification of CCND1 is common in LUSC patients in the TCGA provisional LUSC cohort, that is, 12.7% in Nature 2012, 14.0% Firehose Legacy, 12.4% PanCancer Atlas. **(D)** FGF19 and CCND1 gene amplification were significantly correlated with their respective expressions. **(E)** Linear regression and Pearson correlation of mRNA levels between FGF19 and CCND1 in the TCGA provisional LUSC cohort. Positive correlation between expression of FGF19 and CCND1 was observed. **(F)** Statistical analysis on the individual level based on the TCGA database (http://www.cbioportal.org). **(a)** Amplification and mRNA expression distribution of FGF19 and CCND1. **(b, c)** A 2 × 4 contingency table was generated for 2 categorical variables, FGF19 and CCND1, in 2 observation states, mRNA high and mRNA no alteration (NA); **(b)** Each entry represents the number of observations for a given gene in 1 observation state; **(c)** Entry number of observations of FGF19 and CCND1 in 1 observation state under the premise of co-amplification. **(G)** Positive correlation between FGF19 and CCND1 protein expression levels in the 16 LUSC tissue samples. Pearson χ^2^ test, P <0.001. Scale bar: 100 µm.

We previously reported that FGF19 was frequently amplified (9/37, 24.3%) in smoking LUSC patients (8, 16). The oncogene CCND1, which is neighboring to FGF19 on the chromosome, was also highly amplified (7/37, 18.9%) in smoking LUSC and exhibited significant co-amplification with FGF19 (5/37, 13.5%) (**Figure 1A**). We also analyzed the copy number amplification of FGF19 and CCND1 in LUSC from the Oncomine database and the co-amplification of these two genes in the cBioPortal database. Consistently, copy numbers of FGF19 and CCND1 were increased compared with the normal lung tissues in the TCGA Lung 2 datasets and the Weiss Lung datasets (**Figure 1B**). In addition, FGF19 and CCND1 showed significant co-amplification in the TCGA datasets (Nature 2012, Firehose Legacy, PanCancer Atlas) (**Figure 1C**). This co-amplification could play an important role in the tumorigenesis of LUSC. We further examined whether FGF19 and CCND1 gene amplification promoted their own expression in LUSC. Indeed, the increased expression of FGF19 and CCND1 mRNA in LUSC corresponded to the amplification (**Figure 1D**), and these two genes showed significant co-expression (**Figure 1E**). To further confirm the correlation between gene amplification and expression, we performed statistical analysis on the individual level in the dataset (**Figure 1F**). We observed that the expression of FGF19 and CCND1 was significantly correlated with the amplification of the two genes at the individual level (**Figure 1Fa**) and the ratios of mRNA high for FGF19 and CCND1 given gene amplifications were significantly higher than those calculated in the global view [FGF19: 10.16% (= 8.47% + 1.69%) vs. 3.86% (= 1.50% + 2.36%), CCND1: 66.1% (= 8.47% + 57.63%) vs. 13.52% (= 1.50% + 12.02%)] (**Figure 1Fc** vs. **Figure 1Fb**). Furthermore, we noticed that under the background of FGF19 mRNA high, the CCND1 mRNA high rate was 83.3% (5/6) (**Figure 1Fc**). We assumed that the increased expression of CCND1 in LUSC was related to its amplification, and might also be related to high FGF19 expression. Immunohistochemical staining of 16 LUSC clinical samples also showed that the expression of CCND1 was significantly correlated with the expression of FGF19 (**Figure 1G**). Together, these findings have indicated that FGF19 and CCND1 are co-amplified and co-expressed in human LUSC.

### FGF19 Enhances the Expression of CCND1 *via* FGF19–FGFR4–ERK1/2 Axis in LUSC

**Figure 2:**
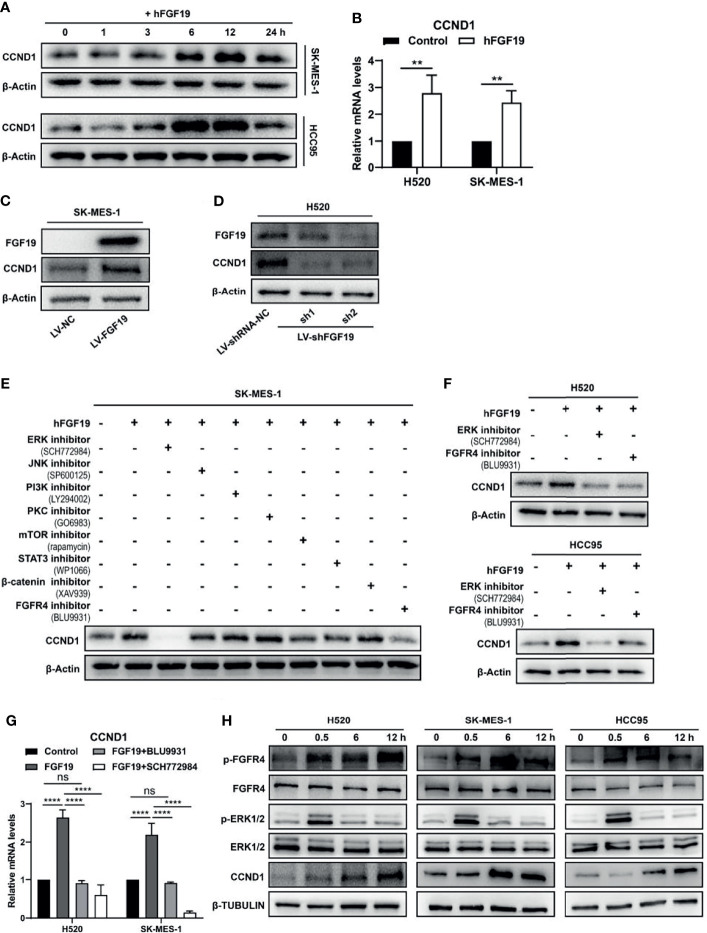
FGF19 enhances CCND1 expression by FGF19-FGFR4-ERK1/2 axis in LUSC cells. **(A)** Recombinant human FGF19 (rhFGF19) (25 ng/ml) promoted expression of CCND1 in SK-MES-1 and H520 cells (serum starved for 12 h before treatment) in a time-dependent manner. **(B)** CCND1 mRNA expression levels in LUSC cell lines after treatment with rhFGF2 for 12 h. **(C)** Expression of CCND1 in FGF19 overexpression LUSC cell line. **(D)** Expression of CCND1 in FGF19 knockdown LUSC cell line. **(E)** A panel of inhibitors against a number of signaling pathways was used to dissect the leading factors of regulated by FGF19. **(F)** Western blot and **(G)** qPCR analysis of CCND1 expression in H520 and HCC95 LUSC cells after treatment of FGF19, FGF19 and SCH772984, FGF19 & BLU9931, or DMSO as control. **(H)** Effects of the FGF19/FGFR4 pathway on protein levels of CCND1, p-FGFR4, and p-ERK1/2 by western blot analysis. H520, SK-MES-1 and HCC95 cells were treated with FGF19 (25 ng/ml, 0/0.5/6/12 h) to activate the FGF19/FGFR4 signaling pathway. Data were shown as mean ± SD bars and compared by unpaired *t*-test. **p <0.01; ****p <0.0001; ns, not significant.

A number of growth factors are known to regulate CCND1 protein production (29, 30), and our analysis of TCGA database also found that under the background of FGF19 mRNA high expression, the frequency of CCND1 mRNA high expression was elevated (**Figure 1Fc**). We investigated the effect of FGF19 on CCND1 expression in LUSC cells by treating with recombinant human FGF19 (rhFGF19). rhFGF19 significantly increased the protein levels of CCND1 in a time-dependent manner, and the highest expression of CCND1 was at the 12-hour time point (**Figure 2A**). Meanwhile, we also verified that rhFGF19 could significantly upregulate the CCND1 mRNA level (**Figure 2B**). Furthermore, FGF19 stably overexpressed LUSC cell line showed increased CCND1 expression (**Figure 2C**). Correspondingly, in the H520 cell line with a high basal level of FGF19, CCND1 expression decreased after FGF19 was knocked down (**Figure 2D**). Together these data confirmed that FGF19 could significantly promote the expression of CCND1.

To further reveal the regulatory mechanism of CCND1 by FGF19, we referred to the classic pathways regulated by FGF19 in the previous research (31) and used a panel of inhibitors against a number of signaling pathways to dissect the leading factors. The ERK1/2 inhibitor SCH772984 completely eliminated the expression of CCND1 even in the presence of rhFGF19 while and the FGFR4 inhibitor BLU9931was also effective to a lesser extent (**Figure 2E**). Because FGF19 is a specific ligand of FGFR4 (32, 33), these results suggested a FGF19–FGFR4–ERK1/2 axis in regulating CCND1. This is also supported by the KEGG database analysis (https://www.kegg.jp/), in which growth factors such as EGF, TGF, and HGF promote the expression of CCND1 through Ras–MEK–ERK axis in non-small cell lung cancer (NSCLC) (**Supplementary Figure 1**). For further verification, two other LUSC cell lines (H520 and HCC95) were used. FGFR4 inhibitor BLU9931 and ERK1/2 inhibitor SCH772984 could markedly inhibit the increase of CCND1 mRNA (**Figure 2G**) and protein (**Figure 2F**) expression caused by FGF19. The experiment with the addition of rhFGF19 also showed that FGF19 could significantly upregulate p-FGFR4, p-ERK1/2, and CCND1 levels, in a clear chronological relationship (**Figure 2H**). To summarize, these observations indicated that the FGF19–FGFR4–ERK1/2 axis significantly promoted the expression of CCND1 in LUSC.

### FGF19 Promotes CCND1-Induced Inactivation of RB

**Figure 3:**
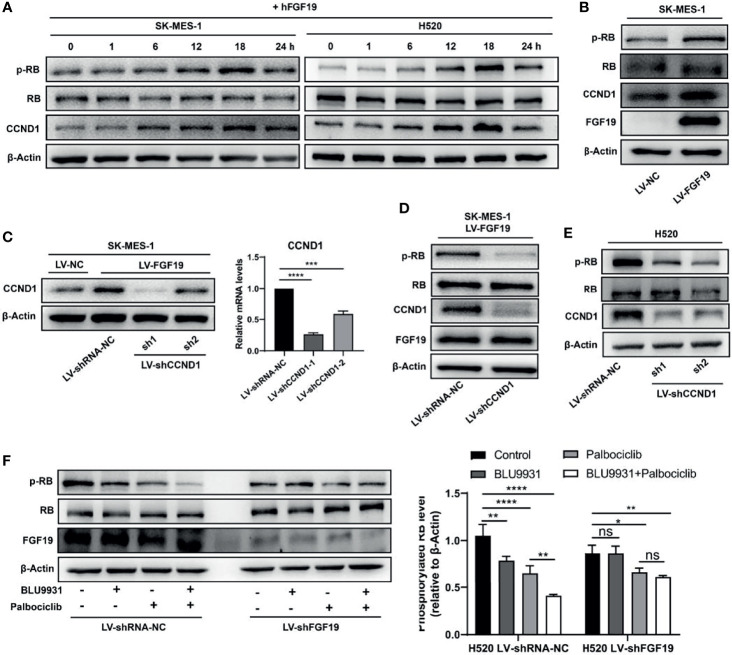
FGF19 enhances CCND1-induced inactivation of RB. **(A)** rhFGF19 phosphorylated RB and promoted expression of CCND1 in SK-MES-1 and H520 cells in a time-dependent manner. **(B)** pRB and CCND1levels in FGF19 overexpression (SK-MES-1 LV-FGF19) and control (LV-NC) LUSC cells. **(C)** SK-MES-1 LV-FGF19 cells was transduced with CCND1-knockdown lentivirus (LV-shCCND1), or control lentivirus (LV-shRNA-NC) to construct stable cell lines and quantification of CCND1 in forms as cellular protein and mRNA. **(D)** pRB and CCND1 levels in FGF19 overexpression and CCND1-knockdown cells (SK-MES-1 LV-FGF19-shCCND1) and control (SK-MES-1 LV-NC-shRNA-NC) LUSC cells. **(E)** pRB and CCND1 levels in CCND1 knockdown cells (H520-shCCND1) and control (H520-shRNA-NC) LUSC cells. **(F)** pRB levels in FGF19-knockdown cells (H520-shFGF19) and control (H520-shRNA-NC) LUSC cells, treated with BLU9931, palbociclib, BLU9931 & palbociclib, or DMSO. Right panel: quantifications of pRB. All the data were shown as the mean ± SD. *P <0.05; **P <0.01; ***p <0.001; ****p <0.0001; ns, not significant.

CCND1, as an important oncogene, has been reported in a variety of cancers (22, 29). It forms a complex with CDK4/6 to promote the phosphorylation of retinoblastoma (RB) gene, which leads to unrestrained E2F transcription factors and promotes cell cycle progression and the malignant proliferation of tumor cells (18–21). To verify whether FGF19 induces the phosphorylation of RB, we treated LUSC cells with rhFGF19 for different durations and examined the level of pRB. Indeed, FGF19 could significantly increase the phosphorylation level of RB (**Figure 3A**). Moreover, the FGF19 stably overexpressed cell line also had a higher level of pRB compared to the control cell line (**Figure 3B**). To verify whether FGF19 regulates the pRB level by CCND1, we knocked down CCND1 in SK-MES-1 LV-FGF19 (SK-MES-1 LV-FGF19-shCCND1) (**Figure 3C**), and found that the pRB expression level of SK-MES-1 LV-FGF19-shCCND1 significantly decreased (**Figure 3D**). Consistently, when CCND1 was knocked down in H520, the level of pRB also decreased (**Figure 3E**). Additionally, in H520 LV-shRNA-NC cells, both FGFR4 inhibitor BLU9931 and CDK4/6 inhibitor palbociclib could significantly inhibit pRB level showing a synergistic effect while in H520 LV-shFGF19 cells, BLU9931 had almost no inhibitory effect on the pRB expression. However, palbociclib could still inhibit the pRB level and the inhibitory level was equivalent to the combined action of these two inhibitors (**Figure 3F**). Collectively, the above data indicated that FGF19 could increase the phosphorylation level of RB by enhancing the expression of CCND1.

### FGF19 Functions in Synergy With CCND1 to Promote Cell Cycle Progression

**Figure 4:**
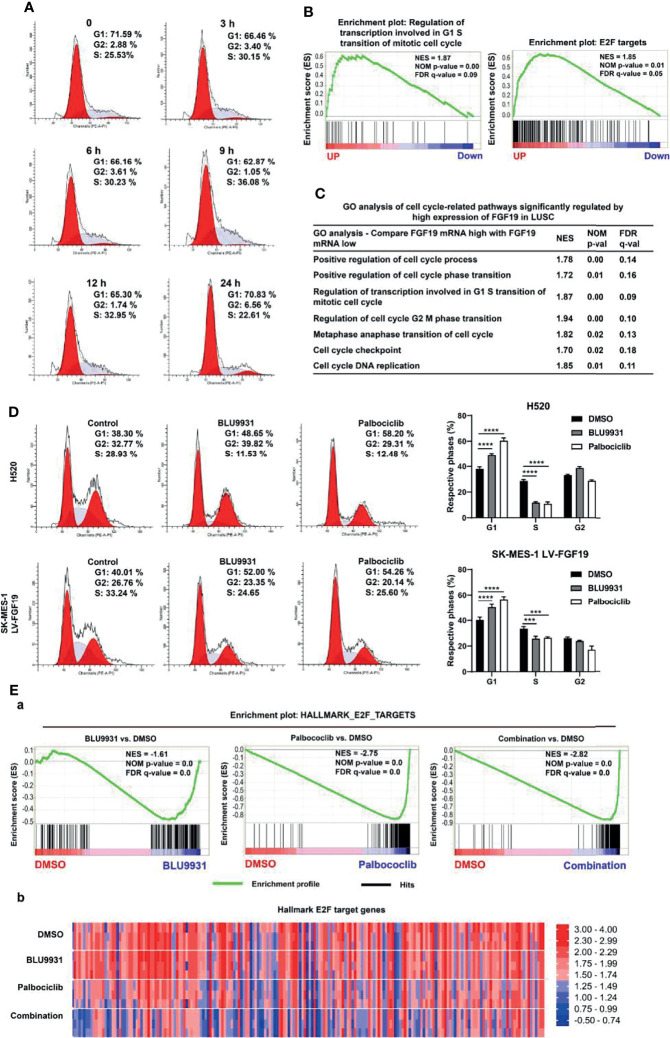
FGF19 combined with CCND1 promotes cell cycle progression. **(A)** rhFGF19 promoted the cell cycle of LUSC cells from G1 to S phase. **(B)** Enrichment plots of regulation of transcription involved in G1/S transition of mitotic cell cycle and E2F targets signatures according to FGF19 expression levels in an LUSC cohort. **(C)** GO analysis of cell cycle-related pathways significantly regulated by high expression of FGF19 in LUSC cohort. **(D)** Representative histograms depicting cell cycle profiles of SK-MES-1 LV-FGF19 and H520 cells with or without BLU9931 and palbociclib. Right panel: quantifications of the histograms. **(E)** H520 was treated with DMSO, 1 μM BLU9931, 1 μM palbociclib or their combination. **(a)** Enrichment plot showing HALLMARK_E2F_TARGETS signatures and **(b)** Heatmap showing the expression of common E2F target genes according to the drug treatment. All the data were shown as the mean ± SD. ***p <0.001; ****p <0.0001.

The abnormal progression of the cell cycle plays a key role in the occurrence and development of cancer (34, 35). Our analysis indicated that the amplification of CCND1 was significantly related to its expression, and FGF19 could further promote the expression of CCND1. High expression of CCND1 promotes the loss of RB, and the inactivation of RB is closely related to cell cycle disorders (29). We thus propose that abnormal expression of FGF19 and CCND1 could significantly promote the progression of the cell cycle, leading to the malignant proliferation of LUSC cells. First, we treated LUSC cells with rhFGF19, and rhFGF19 promoted the cell cycle from G1 to S phase (**Figure 4A**). We then performed a Gene Set Enrichment analysis (GSEA) enrichment analysis in the LUSC dataset. GSEA plots for FGF19 in the LUSC cohort revealed that the regulation of transcription involved in G1/S transition of mitotic cell cycle and E2F targets were enriched in “FGF19 Up” genes (**Figure 4B**), accompanied by other cell cycle-related pathways such as cell cycle checkpoint, cell cycle G2/M phase transition, cell cycle metaphase/anaphase transition, and cell cycle DNA replication (**Figure 4C**). In the profile of the running ES score & positions of geneset members on the rank ordered list, we also observed that CCND1 played a core enrichment position in FGF19 promoting the cell cycle transition of LUSC cells (**Supplementary Figure 2**). Through the STRING database analysis, we found that the co-expression of FGF19 and CCND1 could affect the expression of other cell cycle-related proteins, such as CDK2/4/6, which were also essential for cell cycle regulation (**Supplementary Figure 3A**). Such observations suggested that FGF19 might regulate these proteins in LUSC. Through experiments, we observed that FGF19 upregulated the expression of CDK2, CDK4, and cyclin E while downregulated cell cycle progression inhibitory proteins P21 and P27 (**Supplementary Figure 3B**). Conversely, we demonstrated that both the FGFR4 inhibitor BLU9931 and the CDK4/6 inhibitor palbociclib could significantly arrest the cycle of LUSC cells from G1 to S phase (**Figure 4D**). To evaluate the synergy of FGF19 and CCND1 on cell cycle progression, we performed sequencing analysis of H520 cells following treatment of 1 μM BLU9931 and 1 μM palbociclib (either alone or in combination) for 3 days. GSEA identified that HALLMARK E2F TARGETS were consistently downregulated in both mono-drug treatment and combination treatments (**Figure 4Ea**). Our gene expression analysis also revealed that the combination therapy resulted in stronger repression of the RB-E2F target genes than either single agent treatment consistent with a role of cooperative cell cycle inhibition (**Figure 4Eb**). Overall, these data highlight the existence of crosstalk between FGF19 and cyclin D1-CDK4/6 signaling in LUSC.

### CCND1 Is Required for FGF19-Induced Proliferation

**Figure 5:**
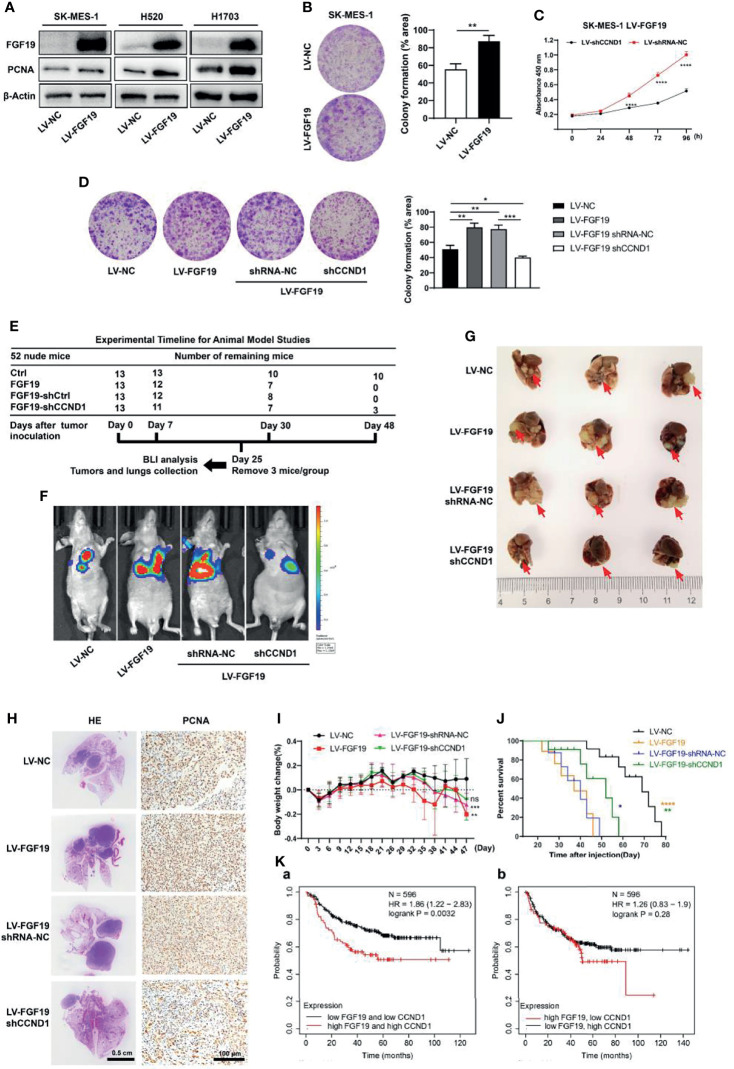
CCND1 is essential for FGF19 signaling-mediated LUSC proliferation. **(A)** Western blot analysis showing protein levels of FGF19 and PCNA in SK-MES-1, H520 and H1703 cells after lentivirus transfection. **(B)** Clone formation assay of SK-MES-1 cells with or without FGF19 overexpression. **(C)** Cell proliferation was measured by CCK8 assay in SK-MES-1 LV-FGF19 cells with or without CCND1 knockdown. **(D)** Clone formation assay of SK-MES-1 LV-FGF19 cells with or without CCND1 knockdown; cultures were stained with crystal violet. **(E–J)** Data of orthotopic lung cancer model. Cell suspension of SK-MES-1 LV-NC/LV-FGF19/LV-FGF19-shRNA-NC/LV-FGF19-shCCND1 (2 × 10^6^ cells) in a total volume of 50 μL mixed with Matrigel (Matrigel: PBS = 1: 4) were injected into the left lung of 5-week-old male BALB/C nude mice (N = 13 mice per group). **(E)** Experimental timeline for the animal experiment. **(F)** Representative bioluminescent images (BLI) of the different groups are shown 25 days after orthotopic implantation. Right panel: quantifications of the total flux. **(G)** Comparison of orthotopic lung cancer models. Tumor was indicated by the arrow. **(H)** Representative H&E and PCNA staining images of lung samples from each group. **(I)** Body weight change and **(J)** overall survival time of indicated groups of nude mice were showed. **(K)** Higher FGF19 and lower CCND1 mRNA levels are associated with longer overall survival. Data were showed as the mean ± SD. *p <0.05; **p <0.01; ***p <0.001; ****p <0.0001. H&E, hematoxylin and eosin.

As observed, FGF19 upregulated PCNA (a marker of cell proliferation) expression (**Figure 5A**), increased cell viability (**Supplementary Figures 4A, B**), promoted colony formation (**Figure 5B** and **Supplementary Figure 4C**) and enhanced Ki67 expression (**Supplementary Figure 4D**) in LUSCs. To determine whether CCND1 was required for FGF19-induced cell proliferation, we investigated the activity of SK-MES-1 LV-FGF19-shCCND1. Knockdown of CCND1 significantly reduced SK-MES-1 LV-FGF19 cells activity (**Figure 5C**). In contrast, overexpression of CCND1 in SK-MES-1 significantly increased cell viability (**Supplementary Figure 4E**). These results indicated that the CCND1 played a critical role during FGF19-induced LUSC cell proliferation. Further, FGF19 overexpression promoted the proliferation of LUSC cells while CCND1 knockdown rescued this process by colony formation assay (**Figure 5D**).

Next, experiments using *in vivo* orthotopic lung cancer model showed that the nude mice implanted with SK-MES-1 LV-FGF19 cells presented increased bioluminescent imaging (BLI) signals, augmented tumor volume in the lung, and significantly increased PCNA expression, resulting in a shorter overall survival time. However, downregulation of CCND1 abrogated the enhanced proliferation ability of SK-MES-1 LV-FGF19 xenograft group, showing reduced BLI signals, lung tumor volume and PCNA expression, resulting in an extended survival time (**Figures 5E**–**J**). Additionally, higher FGF19 and CCND1 mRNA levels (top 25%) were associated with shorter progression free survival. However, there was no significant difference in progression-free survival statistics for higher FGF19 and lower CCND1 mRNA levels (**Figure 5K**). Together, these results suggested that CCND1 was essential for FGF19-mediated LUSC proliferation.

### BLU9931 Synergizes With Palbociclib to Suppress Tumor Growth in LUSC Cells

**Figure 6:**
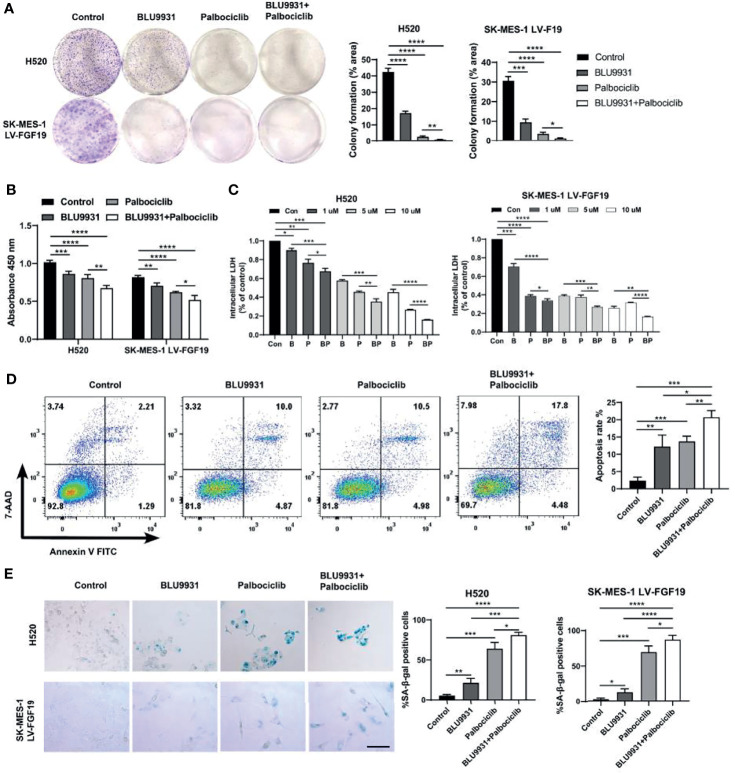
Enhanced effects of combined BLU9931 and palbociclib in LUSC cells *in vitro*. **(A)** Cells were plated at a low density in 6-well plates, treated with DMSO, BLU9931, palbociclib, or a combination of the two compounds for 7–10 days. **(B)** CCK8 cell viability assay, **(C)** Intracellular lactate dehydrogenase (LDH) assay, **(D)** flow cytometry analysis and **(E)** β-galactosidase staining assay of H520 and SK-MES-1 LV-FGF19 cells treated with a single agent (BLU9931 or palbociclib) or a combination of both compounds at a fixed ratio (1:1). *p <0.05; **p <0.01; ***p <0.001; ****p <0.0001; 100 µm.

Based on the co-expression feature of these two genes, dual targeting are expected to be more effective. The combination of BLU9931 and palbociclib significantly reduced the viability and clonogenic potential of H520 and SK-MES-1 LV-FGF19 cells compared with single agent alone (**Figures 6A, B**). To determine whether the anti-tumor effects obtained with different doses of BLU9931 and palbociclib were synergistic or not, we evaluated the combination index. After treatment with various concentrations (1, 5, and 10 μM) of BLU9931, palbociclib, and their combination, the combination index was measured for each cell line. We observed that different doses of inhibitors had a synergistic effect, and the inhibitory effect increased significantly with the increase of the dose (**Figure 6C**). An analysis of apoptosis was also performed, and when compared with the single agent, the combination treatment for 48 h significantly increased the cell apoptosis rate (**Figure 6D**). It has been reported that decreased cell proliferation coupled with cell cycle arrest was associated with senescence induction (36). Therefore, we investigated the senescence-associated SA-β-gal activity after the inhibitors treatment in LUSC cells. SA-β-gal-positive cells increased more markedly in combination treatment when compared to those under single drug treatment or control cells (**Figure 6E**). Moreover, the combination of the two inhibitors showed a stronger inhibitory effect on the expression of RB-E2F target genes than single-drug treatment (**Figure 4E**). Overall, these results suggested that the combination of BLU9931 and palbociclib exerted a synergistic therapeutic effect on FGF19-driven LUSC *in vitro*.

### BLU9931 Synergizes With Palbociclib to Suppress Tumor Growth *In Vivo*

**Figure 7:**
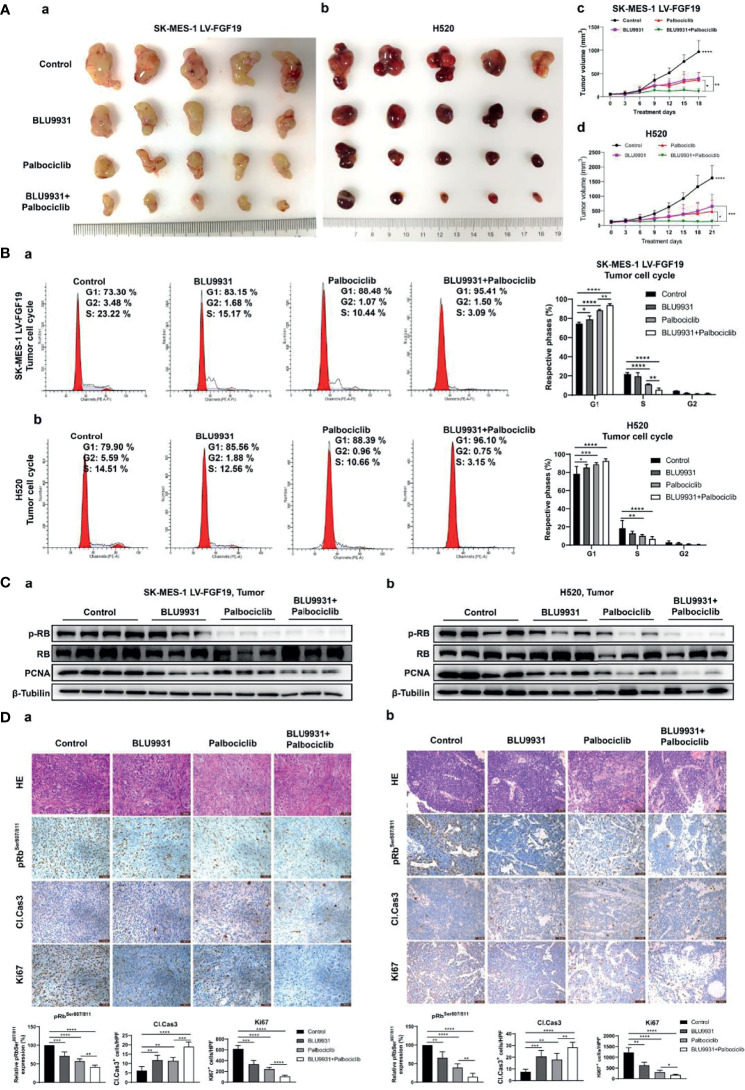
Enhanced effects of combined BLU9931 and palbociclib in the animal model of LUSC cells with endogenously or exogenously high expression of FGF19. SK-MES-1 LV-FGF19 (exogenous FGF19 highly expressed LUSC cells) cells or H520 (endogenous FGF19 highly expressed LUSC cells) cells in a volume of 50 μl were subcutaneously injected into the right flanks of BALB/c nude mice. When the tumor reached a volume of 100–200 mm^3^, mice were randomly grouped and orally treated with vehicle, BLU9931 (30 mg/kg, twice a day), palbociclib (100 mg/kg/d), or both drugs in combination for 3 weeks. Tumor volumes were measured every 3 days after the onset of treatment. **(A)** The tumors were dissected from the mice. **(a)** SK-MES-1 LV-FGF19 xenograft tumors and **(b)** H520 xenograft tumors. Growth curve for **(c)** SK-MES-1 LV-FGF19 and **(d)** H520 xenograft tumors. **(B)** Representative histograms depicting cell cycle profiles of tumor cells and quantifications of the histograms. **(a)** SK-MES-1 LV-FGF19 xenograft tumors; **(b)** H520 xenograft tumors. **(C)** The expression of pRB and PCNA in **(a)** SK-MES-1 LV-FGF19 xenograft tumors and **(b)** H520 xenograft tumors were detected by western blot. **(D)** HE and immunofluorescence staining with pRb^Ser807/811^, cleaved caspase 3 (Cl. Cas3) and Ki67 of tumor sections. **(a)** SK-MES-1 LV-FGF19 xenograft tumors; **(b)** H520 xenograft tumors. Data were shown as mean ± SD and *p <0.05, **p <0.01, ***p <0.001 and ****p <0.0001 as calculated by the two-way ANOVA test. Scale bar: 50 µm.

Since combination treatment showed synergistically inhibitory effect in LUSC cell lines, we investigated whether this similar effect could be recapitulated in the subcutaneous lung cancer mice model. Two cell lines (exogenous or endogenous FGF19 highly expressed LUSC cells) were used to construct animal models. In the mice model of LUSC with exogenous or endogenous overexpression of FGF19, we observed a significant suppression in tumor growth with combination treatment (**Figure 7A**). In the combination group, the median tumor weight of LUSC xenografts was lower when compared to BLU9931, palbociclib and vehicle control (**Supplementary Figures 5A, B**). The inhibitors did not cause a significant decrease in the body weight of the nude mice, and it indicated that the administered dose was within the tolerance range of the nude mice (**Supplementary Figures 5C, D**). BLU9931 and palbociclib monotherapy arrested tumor cells in G1 phase, but the two-drug combination therapy had a stronger response (**Figure 7B**). Meanwhile, the combination therapy had a better inhibitory effect on the levels of phosphorylated RB and PCNA in tumor tissues (**Figure 7C**). Immunohistochemical staining showed that the levels of p-RB (Ser807/811) and of Ki67 in the combined treatment group were significantly lower than those in the control group or single drug treatment group, and a marker of apoptosis, cleaved caspase-3, was increased in the combination treatment tumors (**Figure 7D**). Collectively, these findings demonstrated the enhanced antitumor efficacy in two subcutaneous lung cancer model by co-targeting FGFR4 and cyclin D1-CDK4/6 signaling.

## Discussion

Significant progress has been made in identifying targets and developing therapeutics in advanced LUAD patients, but not for LUSC. While the most substantial impact on the treatment of LUSC patients comes from the histological agnosticism method of immune checkpoint inhibition. A main reason is the failure to find effective molecular targets in LUSC. In this study, we showed that FGF19, which has been well studied in several cancers, was involved in promoting LUSC. Our previous research has demonstrated that FGF19 was upregulated in LUSC tissues and contributed to LUSC progression through the mTOR pathway (16). It was also reported that FGF19 amplification and expression were upregulated in a subset of LUSC patients, and further proved that FGF19 signal activation was necessary for the growth and survival of FGF19-amplified LUSC cells (37). These studies established the important role of FGF19 in the tumorigenesis of LUSC.

In the present study, we have provided more data indicating that FGF19 acts as a tumor promoter in LUSC. Through more in-depth analysis of the previous sequencing data on tumor-promoting amplified genes, in combination with further TCGA data analysis and clinical sample staining verification, we observed that FGF19 expression was positively correlated with CCND1 expression at both the mRNA and protein levels. The expression of the two genes was significantly correlated with their co-amplification on chromosome 11q13.3. Interestingly, our subsequent findings suggested that not only was this co-expression caused by co-amplification, but also FGF19 could regulate the expression of CCND1 at the mRNA and protein expression levels. Gene co-amplification leads to the overexpression of many neighboring genes, some of which can work together and promote the tumor genesis and progression. However, the mechanism of co-amplification gene cooperation varies under different circumstances. It is reported that CDK4 and Phosphoinositide 3-kinase enhancer (PIKE-A) are co-localized on chromosomal 12q13.1-14 and co-amplified in glioblastoma and form a protein complex with each other to promote tumorigenesis (38). Similar mechanism was found for ACTL6A and p63 in head and neck squamous cell carcinoma (7), PRL-3 and FAK in hepatocellular carcinoma (39). Differently, S100A7, S100A8 and S100A9 are co-amplified on chromosome 1q21.3 in breast cancer, have similar functions and cooperate with each other to induce target protein phosphorylation leading to tumor recurrence and chemoresistance (6). There are also some similar reports on non-coding genes (40, 41). Therefore, the role of co-amplification genes related to the tumor promotion process involves different mechanisms. In our data, we identified CCND1 as a significant component of the FGF19 signaling pathway. Mechanistically, our data indicated that the main reasons for the overexpression of CCND1 could be caused by its own amplification or the regulation of FGF19, and likely by the action of compounded effects of these two genes.

The overexpressed CCND1 forms a complex with CDK4/6 to promote phosphorylation and inactivation of RB, which facilitates a release of E2F necessary for cell cycle S phase entry (42). Therefore, targeting RB is considered to be a viable strategy against tumor growth. Palbociclib is the first CDK4/6 selective inhibitor for breast cancer treatment approved by the FDA. Preclinical studies have shown that increased expression of RB and cyclin D1 and deletion of p16 are associated with the effects of palbociclib on breast cancer, lymphoma, sarcoma, and other tumors including lung cancer (43). Our results showed that palbociclib could significantly inhibit the cell cycle progression of FGF19-driven LUSC cells. BLU9931 could decrease the CCND1 expression by inhibiting FGFR4/ERK1/2 signaling and resulting in G1 phase arrest. The two drugs could also inhibit the target gene expression of transcription factor E2F and showed a superior inhibiting effect on the proliferation of tumor cells.

However, it is undeniable that the way in which an oncogene contributes to tumor cell malignancy is often not single. This is also an important reason for drug resistance in the monotherapy. As one of the important growth factors, FGF19 also regulates some other cancer-related pathways. For instance, FGF19-mediated activation of the MAPK, PI3K/AKT, STAT3 and epithelial–mesenchymal transition pathways might take part in the malignancy (44, 45). Our GSEA analysis also revealed that FGF19 enriched many pathways related to cell cycle progression except for positive regulation of G1 to S phase transition. Moreover, the amplification of CCND1 itself could also promote its abnormal expression leading to tumorigenesis. Of note, Lung-MAP (SWOG S1400), a master platform trial for assessing LUSC targeted therapies, showed palbociclib, as a monotherapy for patients with cyclin D (CCND1, CCND2, and CCND3) and CDK4 alterations, failed to prove the prespecified criteria for entering the phase III trial. Despite the results of this study, CCND1 amplification was observed in a subset of responding patients (46). As mentioned previously, palbociclib had little activity as a single drug in the treatment of breast cancer, but it was obviously beneficial to combine it with other drugs (47, 48). In addition, evidence from other tests has indicated that additional evaluation of this pathway might be beneficial to some LUSC patients. Thus, in such condition, dual targeting is a better scenario. Sequencing data analysis has also shown that the combination of these two drugs acted better in the inhibition of the target gene expression of E2F, and in the cell cycle progression arrest. Compared with single-agent therapy, the co-targeting of FGFR4 and CDK4/6 could significantly inhibit the clonal formation of LUSC cells, reduce cell viability, and promote cell death. Apoptosis analysis also showed a synergistic effect of the two drugs. Treatment-induced senescence is a novel method to induce tumor cell cytostasis. BLU9931 and palbociclib significantly increased the number of SA β-galactosidase positive cells. These effects were further enhanced when used in combination indicating dual targeting effectiveness. Our data also showed that co-targeting FGFR4 and CDK4/6 could significantly inhibit tumor growth in both the mouse models.

It is worth mentioning that chromosome region 11q13.3 also contains several other genes, such as CTTN, ORAOV1, MYEOV, etc. Amplification and overexpression of CTTN contribute to the metastasis of cancer cell by promoting cell migration and anoikis resistance (49). ORAOV1 enhances tumorigenicity and is associated with tumor histology through proline metabolism and reactive oxygen species production. Previous studies have shown that ORAOV1 amplification or overexpression is associated with poor prognosis (50, 51). MYEOV is overexpressed in some cancers and contributes to tumorigenesis, metastasis and poor prognosis, including NSCLC (52, 53). It is likely that these genes could also be co-amplified and overexpressed with FGF19 in LUSC, and contribute to the cancer phenotype. However, the impact of these genes could also be different, as several studies have implicated in other cancer types (54, 55). Therefore, whether these genes do play a role in LUSC and whether FGF19 signals interacts with these genes requires further investigation.

**Figure 8:**
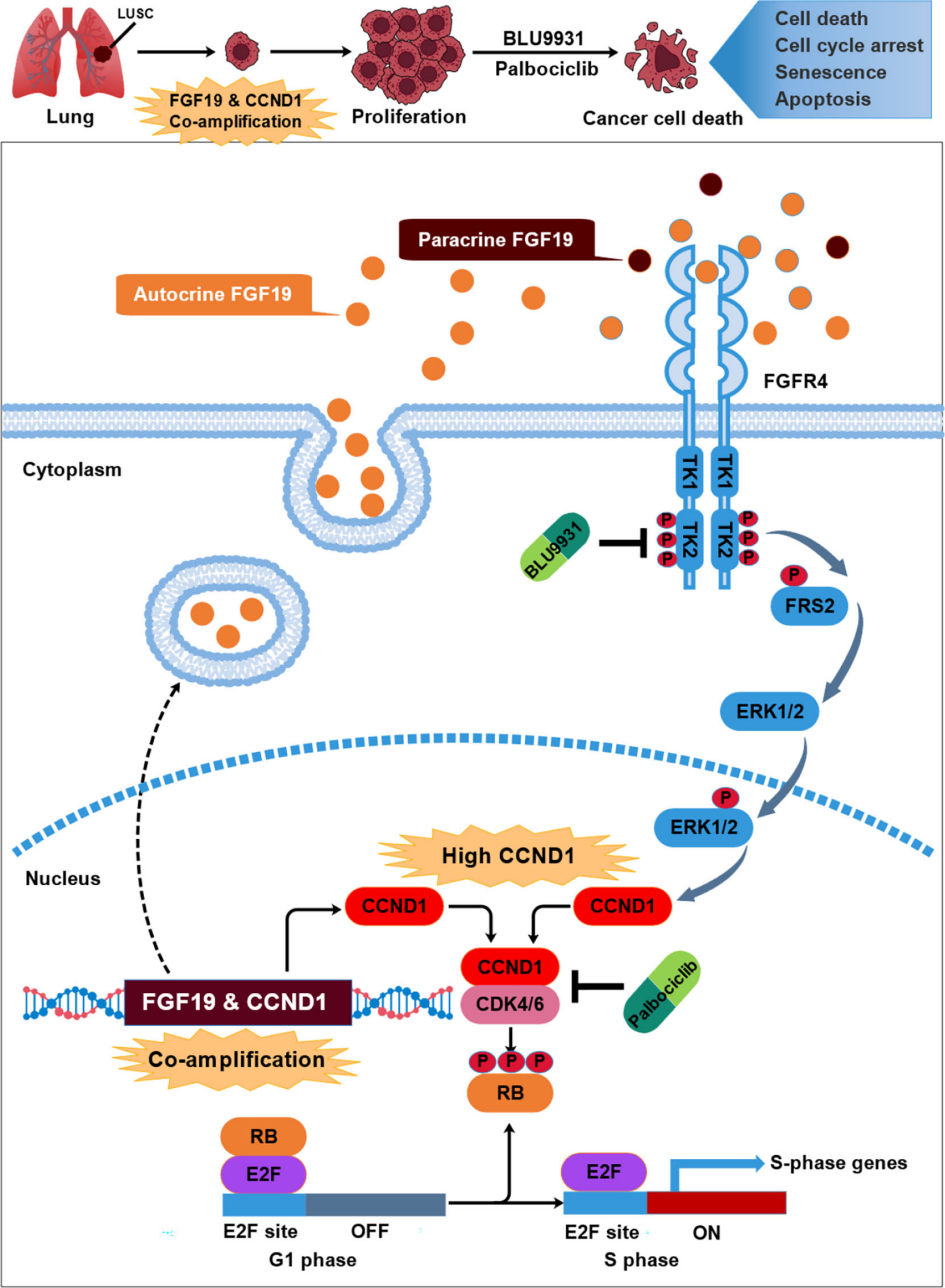
The schematic model of this study: underlying mechanism that link neighboring oncogene FGF19 & CCND1 co-amplification in the development of LUSC. FGF19 was co-amplified and co-expressed with its neighboring gene CCND1 in a subset of LUSC. Overexpression of FGF19 activates FGFR4 and leads to phosphorylation of FRS2 and ERK1/2, which further strengthens the increase in CCND1 expression caused by amplification. These lead to the phosphorylation of RB by CCND1-CDK4/6 complex, causing unrestrained transcription factor E2F, which promotes cell cycle progression and lead to the malignant proliferation of LUSC. While co-targeting FGFR4 and CDK4/6 by BLU9931 and palbociclib potentiated the growth inhibition and arrested cells in G1 phase.

In conclusion, our study provides an example of how co-amplified genes work together in malignant cancer. FGF19 is commonly amplified and overexpressed in LUSC and that CCND1 is co-expressed with FGF19 due to co-amplification on chromosome 11q13.3 in LUSC. FGF19 enhances CCND1 expression, promotes CCND1-induced phosphorylates, and inactivates RB leading to proliferation of LUSC cells, *via* the FGF19–FGFR4–ERK1/2 signaling pathway. Furthermore, combination of BLU9931 with palbociclib potentiated antitumor activities in FGF19-driven LUSC preclinical models. These findings warrant further clinical investigations and patients with advanced LUSC harboring FGF19-CCND1 co-overexpression may therefore benefit from such combination therapy (**Figure 8**).

## Data Availability Statement

The datasets presented in this study can be found in online repositories. The names of the repository/repositories and accession number(s) can be found in the article/**Supplementary Material**.

## Ethics Statement

The studies involving human participants were reviewed and approved by The Research Ethics Committee of the School of Biomedical Engineering, Shanghai Jiao Tong University (Shanghai, China). The patients/participants provided their written informed consent to participate in this study. The animal study was reviewed and approved by The Research Ethics Committee of the School of Biomedical Engineering, Shanghai Jiao Tong University (Shanghai, China).

## Author Contributions

Conception and design: YZ and WX. Data acquisition and analysis: YZ, TW, FL, YC, QH, XL and WX. Writing and original draft preparation: YZ, TW, and WX. Critical review and editing: YZ, TW, YC, and WX. Studies related with clinical samples: SL. Supervision: WX. Funding acquisition: WX. All authors listed have made a substantial, direct, and intellectual contribution to the work and approved it for publication.

## Funding

This study is supported by grants from Science and Technology Commission of Shanghai Municipality (21ZR1433100) and National Natural Science Foundation of China (81773115).

## Conflict of Interest

The authors declare that the research was conducted in the absence of any commercial or financial relationships that could be construed as a potential conflict of interest.

The reviewer WC declared a shared affiliation with the authors to the handling editor at the time of review.

## Publisher’s Note

All claims expressed in this article are solely those of the authors and do not necessarily represent those of their affiliated organizations, or those of the publisher, the editors and the reviewers. Any product that may be evaluated in this article, or claim that may be made by its manufacturer, is not guaranteed or endorsed by the publisher.
