# Supplementary material for: FGF19 Is Coamplified With CCND1 to Promote Proliferation in Lung Squamous Cell Carcinoma and Their Combined Inhibition Shows Improved Efficacy
Source: Front Oncol. 2022 Apr 7;12:846744. doi: 10.3389/fonc.2022.846744 (PMC9021371; doi:10.3389/fonc.2022.846744)
Supplement: Supplementary file 1 [file Image_1.pdf]

Supplementary Information of “*FGF19 is co-amplified with CCND1 to promote proliferation in lung squamous cell carcinoma and their combined inhibition shows improved efficacy*”

Five supplementary figures.

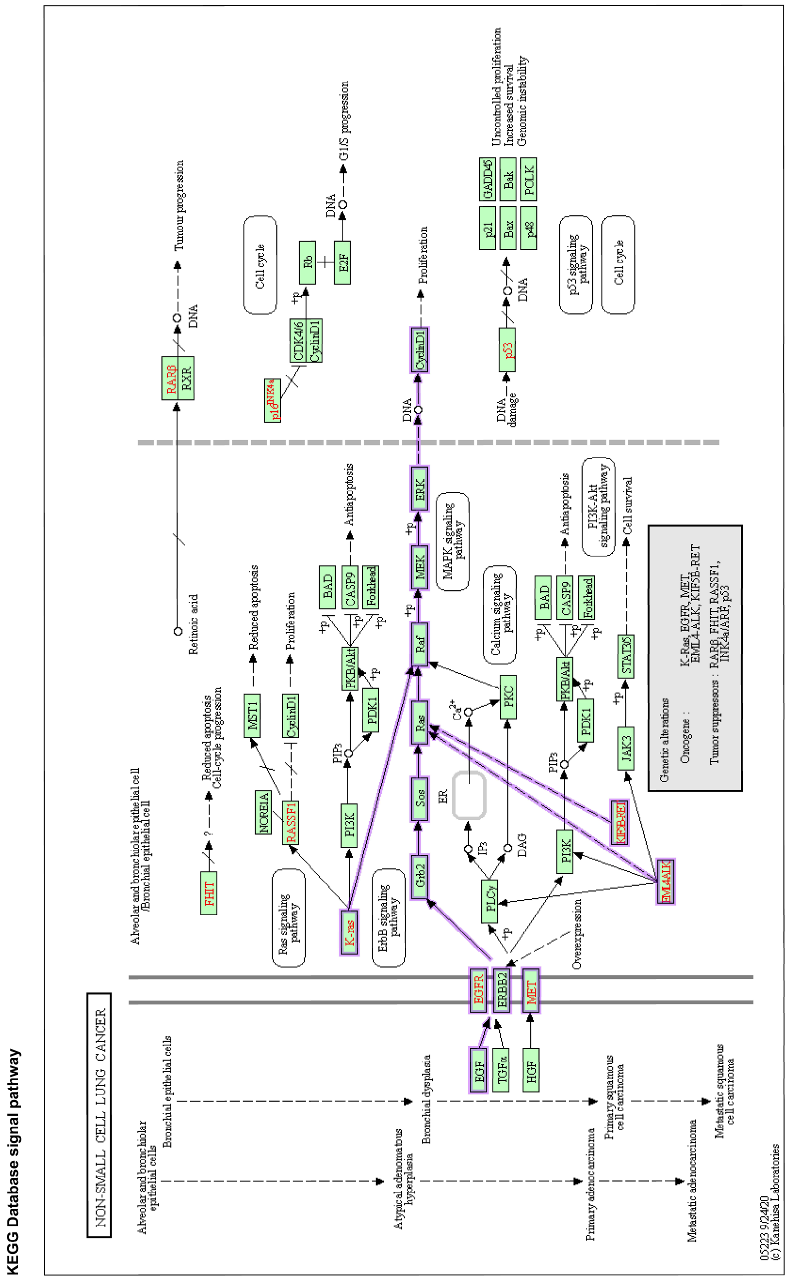

Supplementary Figure S1. Diagram of the KEGG Database signaling pathways. MEK-ERK signaling pathway regulated by the EGF/TGFα/HGF in NSCLC.

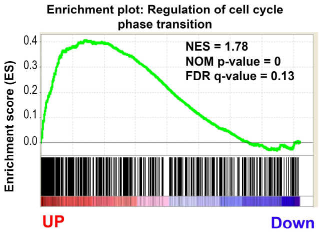

Profile of the Running ES Score & Positions of GeneSet Members on the Rank Ordered List

NAME	RANK IN GENE LIST	RANK METRIC SCORE	RUNNING ES	CORE ENRICHMENT	NAME	RANK IN GENE LIST	RANK METRIC SCORE	RUNNING ES	CORE ENRICHMENT
GLI1	15	0.4553	0.0078	Yes	PSMD14	1285	0.2130	0.3070	Yes
DTL	24	0.4442	0.0158	Yes	MAPRE1	1290	0.2126	0.3108	Yes
PSMD11	34	0.4281	0.0234	Yes	ORC1	1293	0.2123	0.3147	Yes
ZNF207	98	0.3776	0.0273	Yes	CDC25C	1335	0.2095	0.3166	Yes
CDC26	102	0.3762	0.0342	Yes	PHB2	1378	0.2066	0.3183	Yes
CDC14B	111	0.3709	0.0408	Yes	E2F7	1388	0.2060	0.3218	Yes
RFWD3	128	0.3634	0.0468	Yes	CDC73	1399	0.2054	0.3251	Yes
CDC25A	130	0.3631	0.0536	Yes	PAFAH1B1	1401	0.2054	0.3289	Yes
CEP78	134	0.3625	0.0603	Yes	CNOT2	1431	0.2035	0.3313	Yes
NEK2	142	0.3578	0.0666	Yes	ANAPC1	1434	0.2031	0.3350	Yes
KIF14	155	0.3539	0.0727	Yes	AURKA	1447	0.2024	0.3382	Yes
BRCA1	159	0.3525	0.0792	Yes	NPM1	1469	0.2008	0.3410	Yes
HAUS8	176	0.3486	0.0849	Yes	CNTRL	1517	0.1983	0.3424	Yes
BUB1	225	0.3326	0.0888	Yes	DBF4	1521	0.1980	0.3459	Yes
PSMB7	242	0.3281	0.0941	Yes	PSMD4	1603	0.1929	0.3455	Yes
NAE1	243	0.3276	0.1003	Yes	RNASEH2B	1628	0.1913	0.3479	Yes
CDC14C	256	0.3248	0.1058	Yes	SKP1	1630	0.1912	0.3515	Yes
CDK5RAP2	261	0.3241	0.1117	Yes	CNOT11	1663	0.1894	0.3534	Yes
UIMC1	288	0.3182	0.1164	Yes	TFAP4	1668	0.1891	0.3568	Yes
SENP2	299	0.3161	0.1218	Yes	CUL1	1682	0.1885	0.3597	Yes
AURKB	323	0.3095	0.1265	Yes	CDT1	1686	0.1883	0.3631	Yes
ATAD5	356	0.3036	0.1306	Yes	PLK4	1713	0.1872	0.3653	Yes
PSMD2	370	0.3015	0.1356	Yes	FAM83D	1791	0.1841	0.3649	Yes
YWHAE	380	0.3006	0.1408	Yes	CDC27	1811	0.1833	0.3674	Yes
PSMD6	390	0.2991	0.1460	Yes	PSMA3	1961	0.1761	0.3633	Yes
TOPBP1	393	0.2988	0.1515	Yes	TFDP2	1982	0.1747	0.3656	Yes
PSME3	414	0.2952	0.1561	Yes	RAD51C	2011	0.1730	0.3675	Yes
CCND1	426	0.2934	0.1610	Yes	MAD2L1BP	2012	0.1729	0.3707	Yes
CDC6	434	0.2923	0.1662	Yes	CEP41	2026	0.1722	0.3733	Yes
TUBB4B	526	0.2790	0.1669	Yes	PSMB6	2044	0.1709	0.3757	Yes
HAUS7	529	0.2787	0.1720	Yes	PSMA2	2186	0.1653	0.3718	Yes
CDC23	533	0.2783	0.1771	Yes	PCNA	2188	0.1652	0.3748	Yes
ZNF830	541	0.2773	0.1820	Yes	CDC25B	2222	0.1639	0.3762	Yes
HMMR	571	0.2727	0.1856	Yes	BLM	2273	0.1623	0.3768	Yes
CEP76	601	0.2696	0.1893	Yes	APPL2	2336	0.1595	0.3767	Yes
SPDL1	654	0.2647	0.1917	Yes	ESPL1	2374	0.1580	0.3779	Yes
MAD2L1	658	0.2639	0.1965	Yes	MTBP	2418	0.1563	0.3786	Yes
PSMD5	700	0.2592	0.1993	Yes	CDK2	2453	0.1552	0.3799	Yes
ADAM17	737	0.2557	0.2023	Yes	MDM4	2488	0.1543	0.3811	Yes
DYNC1LI1	781	0.2511	0.2049	Yes	BUB3	2518	0.1532	0.3825	Yes
HAUS6	786	0.2508	0.2094	Yes	APPL1	2586	0.1505	0.3820	Yes
NDC80	790	0.2504	0.2140	Yes	EIF4G1	2619	0.1492	0.3832	Yes
CDC45	796	0.2498	0.2184	Yes	CNOT10	2745	0.1455	0.3797	Yes
CCNB1	869	0.2422	0.2194	Yes	BUB1B	2758	0.1452	0.3819	Yes
PSMD3	875	0.2416	0.2237	Yes	RAD21	2783	0.1445	0.3834	Yes
TTK	902	0.2399	0.2269	Yes	RINT1	2831	0.1431	0.3837	Yes
YWHAG	911	0.2391	0.2310	Yes	CNOT6	2832	0.1430	0.3864	Yes
CKAP5	919	0.2387	0.2351	Yes	UBE2E1	2889	0.1414	0.3863	Yes
BRD4	925	0.2381	0.2393	Yes	PSMA1	2893	0.1413	0.3888	Yes
E2F4	926	0.2381	0.2438	Yes	PSMB5	2895	0.1413	0.3914	Yes
PSMB4	944	0.2364	0.2474	Yes	VPS4A	2901	0.1410	0.3938	Yes
CDK1	951	0.2360	0.2515	Yes	HUS1	2945	0.1397	0.3943	Yes
WNT10B	997	0.2329	0.2537	Yes	PAXIP1	2964	0.1393	0.3960	Yes
HMGA2	1004	0.2323	0.2577	Yes	STOX1	2984	0.1388	0.3977	Yes
TPX2	1017	0.2315	0.2615	Yes	ANAPC2	3033	0.1373	0.3979	Yes
PSMD7	1035	0.2304	0.2650	Yes	ID2	3094	0.1358	0.3974	Yes
CENPF	1044	0.2294	0.2689	Yes	HUS1B	3121	0.1349	0.3987	Yes
ODF2	1064	0.2278	0.2722	Yes	SDCCAG8	3139	0.1344	0.4004	Yes
CDCA5	1069	0.2273	0.2763	Yes	DCTN2	3214	0.1319	0.3992	Yes
DBF4B	1156	0.2213	0.2762	Yes	EZH2	3287	0.1295	0.3980	Yes
SMARCD3	1163	0.2209	0.2800	Yes	TPRA1	3343	0.1278	0.3977	Yes
CENPJ	1173	0.2203	0.2837	Yes	SSNA1	3346	0.1277	0.4000	Yes
HSP90AA1	1177	0.2201	0.2877	Yes	CEP57	3365	0.1271	0.4015	Yes
BRD7	1208	0.2180	0.2903	Yes	CEP63	3394	0.1262	0.4024	Yes
LSM11	1213	0.2177	0.2942	Yes	RAD51B	3412	0.1259	0.4040	Yes
GTSE1	1225	0.2170	0.2977	Yes	TM4SF5	3437	0.1251	0.4051	Yes
ERCC3	1234	0.2165	0.3014	Yes	PSMD12	3445	0.1250	0.4071	Yes
PLK1	1274	0.2136	0.3035	Yes					

Supplementary Figure S2. Core enrichment position in FGF19 promoting the cell cycle transition of LUSC cells. Enrichment plots of regulation of cell cycle phase transition signatures according to FGF19 expression levels in an TCGA LUSC cohort (Firehose Legacy). The profile of the running ES score & positions of geneset members on the rank ordered list was shown.

A

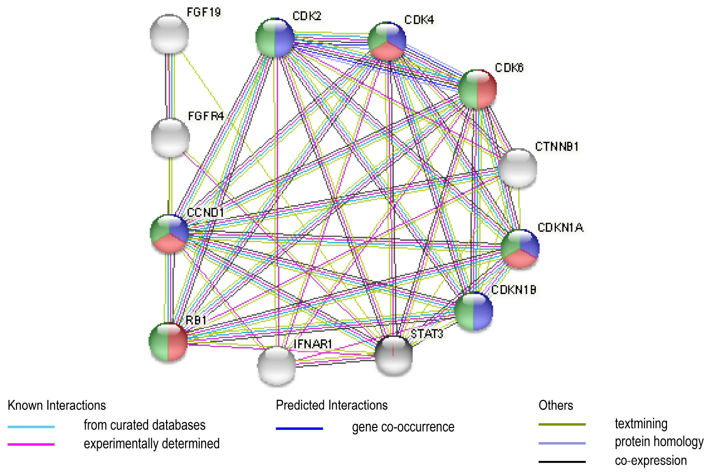

B

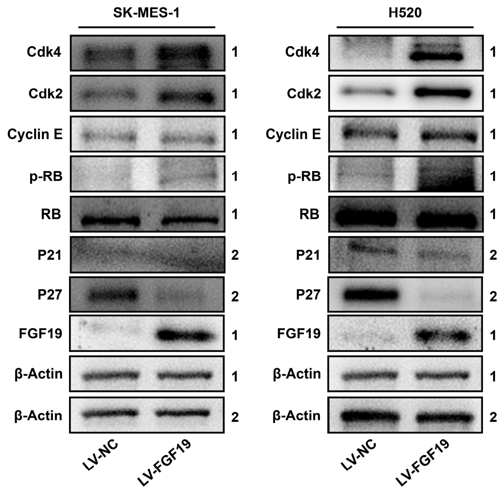

Supplementary Figure S3. Several cell cycle regulatory proteins involved in FGF19 and CCND1 expression. (A) STRING Database (https://string-db.org/) analyzed the genes associated with FGF19 and CCND1 expression. (B) Western blot analysis showing protein levels of CDK2/4, cyclin E, P21 and P27 in SK-MES-1 and H520 cells after FGF19-lentivirus transfection.

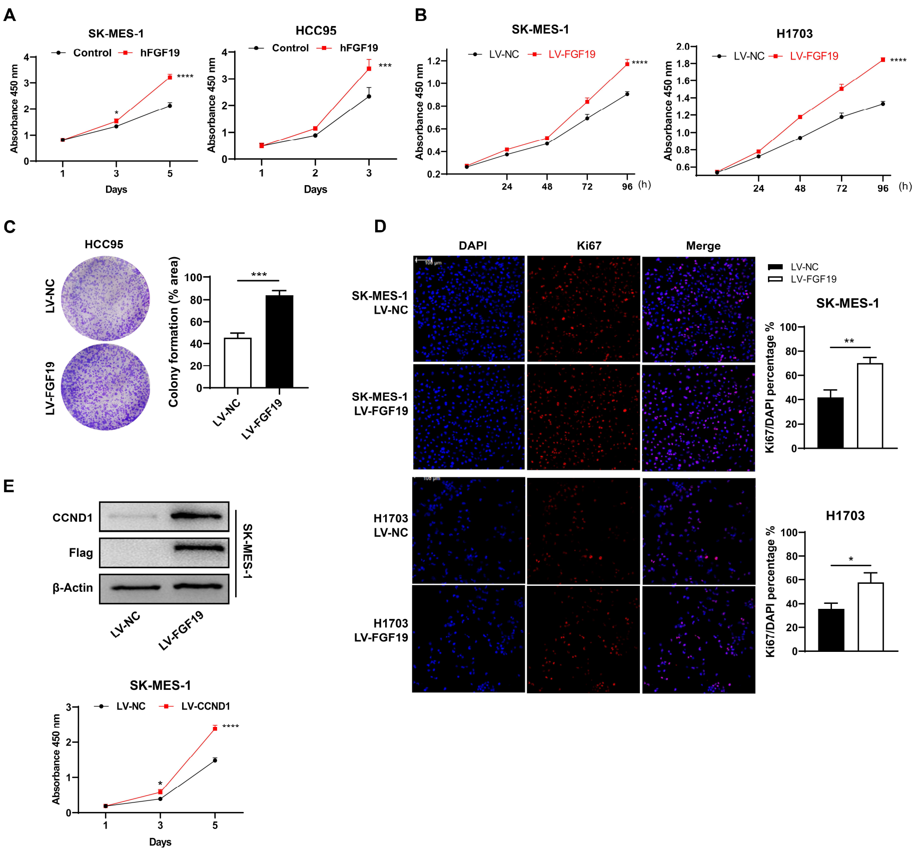

Supplementary Figure S4. Both FGF19 and CCND1 overexpression can significantly promote LUSC cells proliferation. (A-B) Cell proliferation was measured by CCK8 assay (A) in SK-MES-1 and HCC95 cells after rhFGF19 treatment, and (B) in SK-MES-1 and H1703 cells after FGF19-lentivirus transfection. (C) Clone formation assay of HCC95 PCDH/LV-FGF19 cells, cultures were stained with crystal violet. (D) Immunofluorescence assay in SK-MES-1 and H1703 cells afte r FGF19-lentivirus transfection. Scale bar: 100 μm. (E) CCK8 assay in SK-MES-1 cells after CCND1-lentivirus transfection.

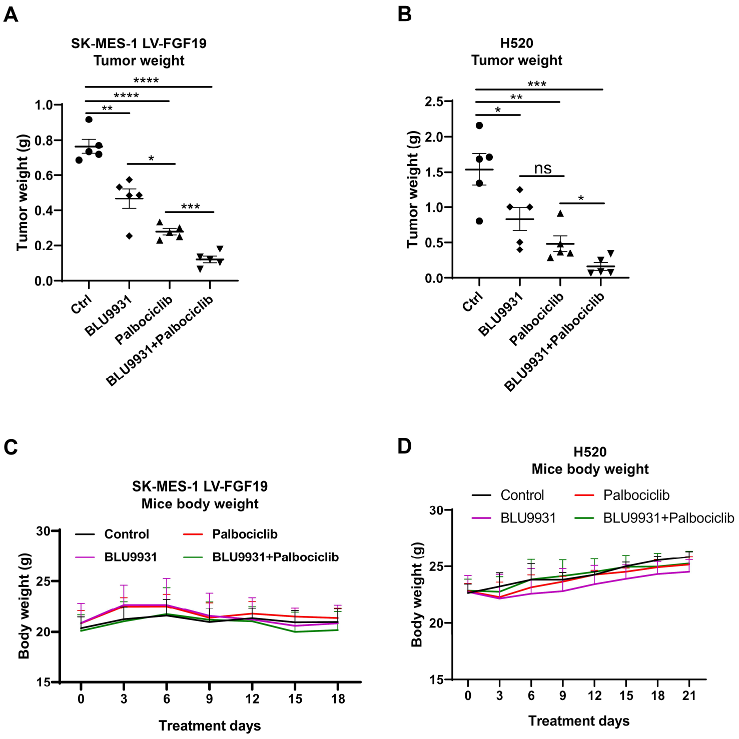

Supplementary Figure S5. Enhanced effects of combined BLU9931 and palbociclib in the animal model of LUSC cells with high expression of FGF19. Tumor weight (**A & B**) and body weight (**C & D**) of mice in each group of SK-MES-1 LV-FGF19 and H520 xenograft tumors were presented.
